# Iatrogenic Bladder Rupture Resulting From the Modified Stoppa Approach for Acetabular Fracture: A Case Report

**DOI:** 10.7759/cureus.66207

**Published:** 2024-08-05

**Authors:** Adnane Lachkar, Hicham Yacoubi, Najib Abdeljaouad

**Affiliations:** 1 Orthopedic Trauma Department B, Mohammed VI University Hospital, Oujda, MAR; 2 Orthopedic Trauma Department B, Faculty of Medicine and Pharmacy of Oujda, University Mohammed First, Oujda, MAR

**Keywords:** bladder, modified stoppa approach, acetabular fracture, iatrogenic bladder injury, iatrogenic complication

## Abstract

Iatrogenic bladder rupture is a rare yet serious complication associated with orthopedic surgical procedures, particularly those involving the modified Stoppa (MS) approach for acetabular fractures. We present a case of a 65-year-old patient who experienced iatrogenic bladder rupture during surgery for acetabular fracture fixation using the MS approach. Despite the challenges posed by this complication, prompt diagnosis and repair during the same surgical intervention led to favorable outcomes. Our case underscores the importance of perioperative vigilance in detecting and managing such injuries to mitigate the risk of urinary tract complications and late infections. Understanding the anatomical nuances and employing meticulous surgical techniques are essential for minimizing the risks associated with the MS approach.

## Introduction

Acetabular fractures are among the most challenging traumas for orthopedic surgeons to manage, especially those involving the anterior column and quadrilateral plate [1]. The modified Stoppa (MS) approach is one of the most commonly used anterior approaches [2]. This approach provides good exposure to the anterior column, the entire quadrilateral surface, and the pubic symphysis [3]. However, it poses some risks and dangers for pelvic organs and the neurovascular bundle [4]. Bladder injury, though very rare and severe, can be unrecognized by orthopedic surgeons if not carefully attended to. We report an exceptional case of iatrogenic bladder rupture diagnosed during the MS approach for acetabular fracture.

## Case presentation

**Figure 1:**
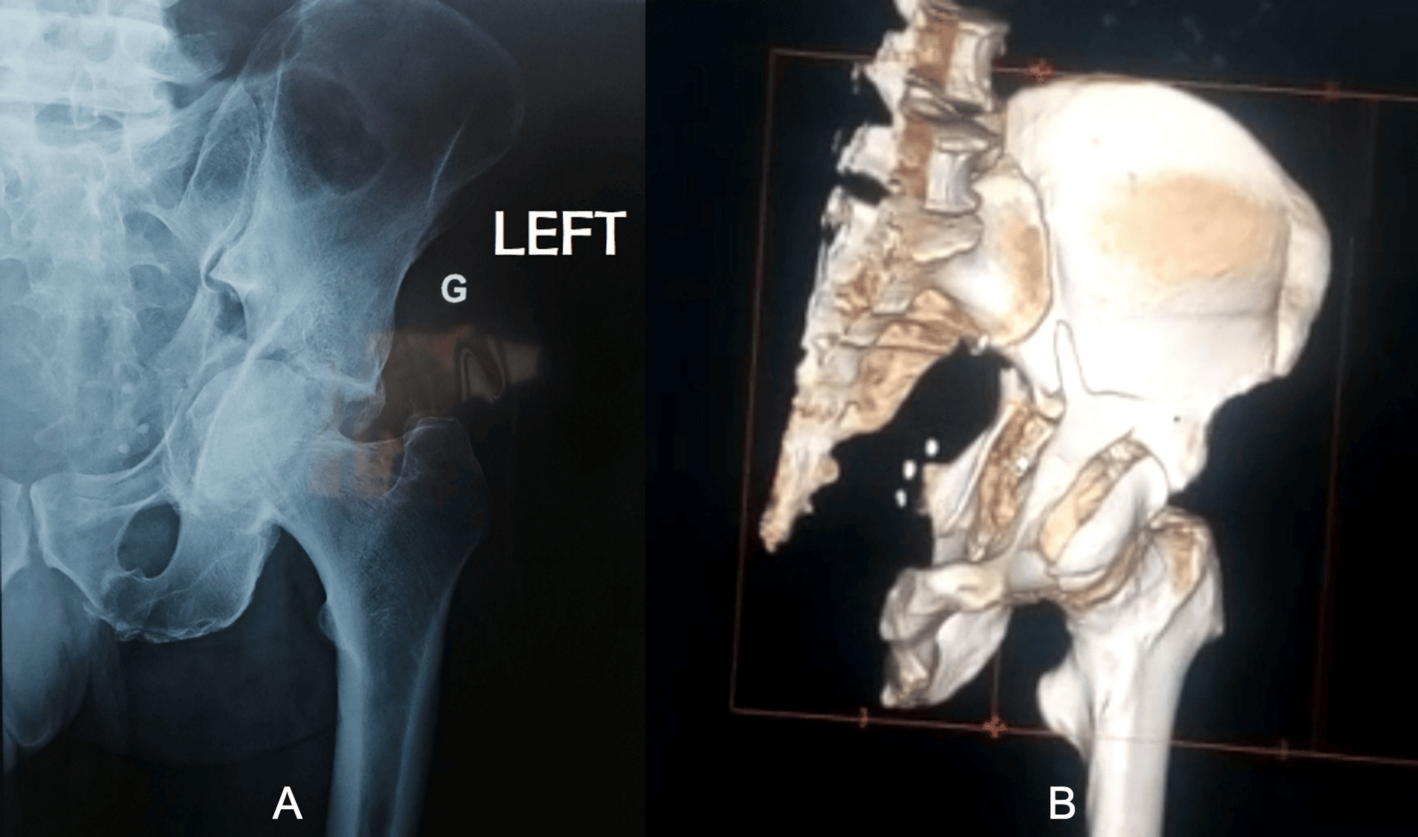
Left acetabular comminuted fracture (anterior column with posterior transverse fracture) (A) Anteroposterior view X-ray of the left hip. (B) 3D CT reconstruction of the fracture

A 65-year-old patient with no previous medical or surgical history presented to the emergency department following a car rollover. He complained of pain and swelling around his left hip and groin with no other complaints. The clinical examination revealed a conscious patient who was hemodynamically and respiratorily stable. The left lower limb was shortened with no ability to stand or bear weight on it. The palpation and mobility of the left hip were excessively painful, and neurological examination was normal. The X-ray and CT scan of the hip showed a comminuted acetabular fracture (anterior column type according to the Judet and Letournel classification) with a displaced quadrilateral surface (Figure 1). The patient received analgesics, and lower limb traction was applied for five days before he was admitted to the operating room.

**Figure 2:**
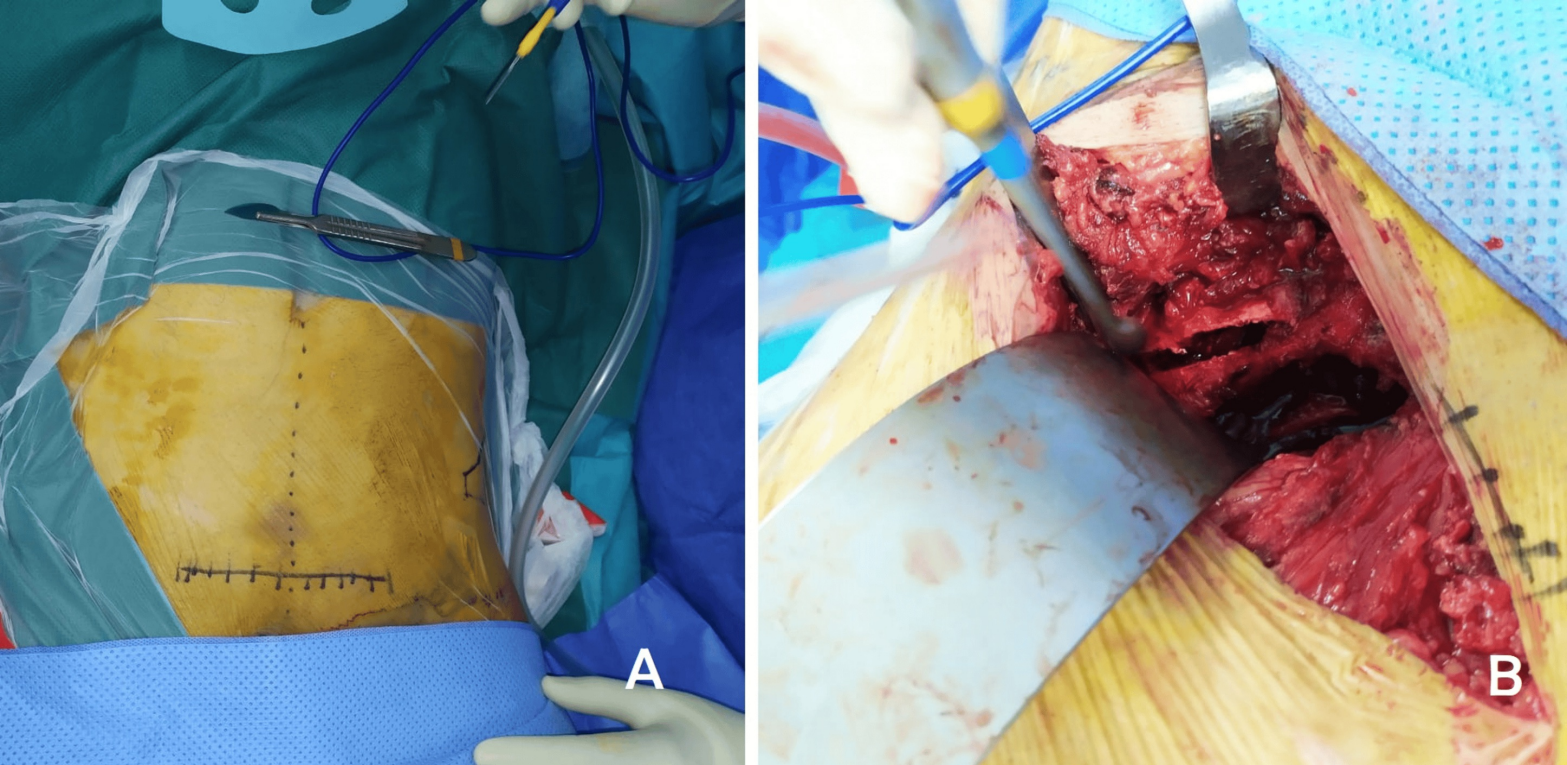
Perioperative views of the modified Stoppa (MS) approach (A) Markings for the Pfannenstiel incision on the skin. (B) Exposure of the fracture using the MS approach with a Leriche retractor placed on the bladder

The patient underwent surgery for open reduction and internal fixation (ORIF) using the MS approach. In the supine position and under general anesthesia, a Pfannenstiel incision was made to expose the rectus abdominis and the linea alba, which were longitudinally divided to access the Retzius space. The medial part of the rectus abdominis was partially detached to allow better exposure of the quadrilateral surface (Figure 2). Special attention was also given to the vascular connections in this approach (corona mortis anastomosis); this anastomosis was identified and ligated before proceeding further with the surgery. A Leriche retractor was applied to retract the bladder and pelvic contents to allow better visualization of the quadrilateral surface.

**Figure 3:**
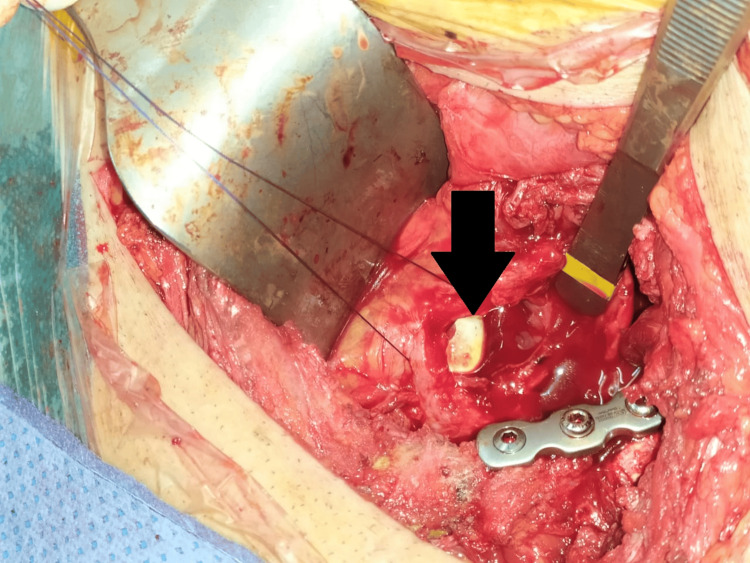
Perioperative view of the bladder injury The arrow indicates the balloon of the Foley catheter

**Figure 4:**
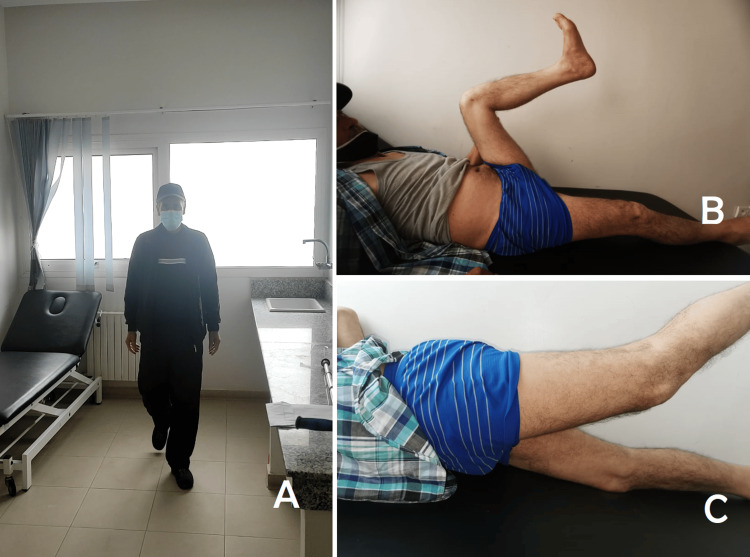
Pictures of the patient at long-term follow-up (two years after the surgery) (A) Walking is possible without limitation or pain. (B) Flexion of the hip at 120 degrees. (C) Extension of the hip at 30 degrees

After reducing and osteosynthesizing the fracture, we discovered a vesical rupture, and the balloon of the Foley catheter (inserted before surgery) was directly visualized (Figure 3). A standard repair technique with two-layer closure using absorbable sutures (Vicryl® 3.0, Ethicon, Inc., Raritan, NJ) was performed. The hospital stay lasted for two days postoperatively. A Foley catheter was left in place for 10 days to decompress the bladder. Postoperatively, there were no complications, and the patient was able to walk at 60 days postoperatively with favorable outcomes. No urine infections or bleeding was reported in the urinary tract, and urination proceeded normally without any complaints. The long-term outcome was favorable, with good functional results according to the Merle d’Aubigne score at the three-year follow-up (Figure 4).

## Discussion

The Stoppa approach was originally described by René Stoppa for inguinal hernia repair [5]. Iatrogenic intraoperative bladder injuries during hernia surgery have been reported in 0.08%-0.3% of cases, with documented incidents in gynecological, general, and urological surgeries [6-9]. In 1994, Cole and Bolhofner [10] introduced the modified Stoppa (MS) approach for treating acetabular fractures involving both the anterior and posterior columns, aiming to reduce the risks associated with the classical ilioinguinal approach. The MS approach provides extensive visual control of the quadrilateral surface, which can present significant challenges for reduction and fixation. However, it remains a technically demanding approach with notable risks and potential hazards for pelvic structures.

To our knowledge, only one case of bladder iatrogenic injury associated with the MS approach has been reported in the literature [11]. Our current case report represents the second documented occurrence. It is well-documented that this approach exposes the bladder to several risks throughout various surgical stages. The primary risk arises during the longitudinal incision of the rectus fascia along the linea alba, where inadvertent bladder or peritoneal injury may occur from the electrosurgical electrode. It is crucial to maintain the entire approach within the pre-peritoneal zone. Identifying the correct line of incision can be challenging for orthopedic surgeons unfamiliar with this surgical region. This line can be more easily identified by digitally palpating the midline of the muscles from proximal to distal parts. We recommend using the lowest possible power setting on the electrocautery when making the incision along the linea alba.

The second risk arises from bladder retractors. Using a Leriche retractor or a malleable retractor that rests on the bladder may lead to laceration or rupture during exposure and reduction maneuvers. We advocate for using a large retractor carefully positioned in the sciatic notch to retract all deep pelvic contents as a single unit, thereby facilitating the exposure of the quadrilateral surface and even the posterior column. To protect the bladder during surgery, we employ a large abdominal swab or gauze to prevent direct contact between the bladder and the retractors.

While this case illustrates a straightforward perioperative diagnosis and a positive long-term functional outcome for our patient, iatrogenic bladder injuries can have significant consequences. Perioperative bladder injuries pose diagnostic challenges and may lead to severe complications, including oliguria, hematuria, and pelvic pain. The patients may also develop abdominal distension, peritonitis, or sepsis if the injury is not promptly recognized [9]. Direct visualization for diagnosis is often challenging, and some authors have suggested using methylene blue instillation, followed by observing pelvic extravasation as a diagnostic approach [9]. In more complex cases, specific radiographic techniques such as CT cystography may be required to confirm the diagnosis.

For their management, extraperitoneal injuries of the bladder are typically treated conservatively with a Foley catheter to decompress the bladder [12]. However, it is reasonable to surgically repair the injury concurrently with osteosynthesis for acetabular fractures.

## Conclusions

Bladder injury during the MS approach for acetabular fractures is a rare but serious complication that must be considered by the orthopedic surgeon. A thorough understanding of pelvic anatomy is crucial to avoid such complications.

The diagnosis of this iatrogenic injury may pose challenges, and careful vigilance is necessary perioperatively to detect it if it arises. Addressing the injury during the same surgical intervention is imperative to mitigate the risk of any urinary tract complications or late infections associated with osteosynthesis.
